# Determinants of pneumonia among under-five children in Oromia region, Ethiopia: unmatched case-control study

**DOI:** 10.1186/s13690-023-01103-5

**Published:** 2023-05-10

**Authors:** Olana Yadate, Aman Yesuf, Fufa Hunduma, Yitagesu Habtu

**Affiliations:** 1 School of Public Health, St. Paul’s Hospital Millennium Medical College, Addis Ababa City Administrative, Addis Ababa, Ethiopia; 2 Department of Public Health, Hossana College of Health Sciences, Hossana City Administrative, Southern Ethiopia Ethiopia; 3 School of Public Health, Department of Preventive Medicine, Addis Ababa University, Addis Ababa City Administrative, Addis Ababa, Ethiopia

**Keywords:** Pneumonia, Acute respiratory infection, Unmatched case-control study, Children under five, Determinants of pneumonia

## Abstract

**Background:**

Pneumonia is the single largest infectious disease that causes more under-five morbidity and mortality than any other infectious disease in the world, including Ethiopia. The aim of this study is to assess determinants of pneumonia among under-five children in the South West Shewa Zone, Oromia Region, Ethiopia, 2021.

**Methods:**

We used an unmatched case-control study design from March 15 to April 30, 2021, in the South West Shewa Zone, Ethiopia. A sample of 398 (199 cases and 199 controls) participated in the study. Trained data collectors through a pre-tested structured questionnaire collected data. We used Epi Info to enter data and analyzed using SPSS version 23. We described our data using descriptive statistics. We identified predictors of pneumonia using logistic regression analysis. We declared predictors of pneumonia at a P-value of 0.05 or less.

**Results:**

Breastfeeding for less than 6 months [AOR:3.51, 95%CI:(1.12,11.00)], lack of Vitamin A supplementation [AOR:3.56,95%CI:(1.58, 8.05)], history of URTI [AOR:9.66, 95%CI:(4.69,19.87)], family child care practices [AOR:6.46, 95%CI, (2.83,14.76)], sleeping with three to five persons in a room [AOR:2.90, 9%CI: (1.23,6.84)], having above five persons in a room [AOR: 3.88, 95%CI: 1.02,14.77), use of wood as a source of fuel [AOR = 3.02 95% CI: 1.41,6.46)] and not opening windows [AOR:2.56 95%CI: (1.21,5.41)] were independent factors of pneumonia among under five children.

**Conclusion:**

Pneumonia is associated with breastfeeding for less than 6 months, lack of vitamin A supplementation, history of URTI, types of childcare practice, indoor overcrowding, use of wood as a source of fuel, and not opening windows. Therefore, exclusive breastfeeding, improving vitamin A supplementation, early control of respiratory tract infection through promoting good hygiene and ventilation strategies in crowded homes, and promoting how to reduce indoor air pollution through affordable clean stoves will be relevant interventions to reduce under-five pneumonia.

## Background

Pneumoniae is a form of acute respiratory infection that affects the lungs and can be caused by pathogens like viruses, bacteria or fungi. When a person gets pneumoniae, the alveoli (small sacs that make up the lungs) are filled up with pus and fluid, making breathing difficult and limiting oxygen intake [1]. The bacterial pathogen Streptococcus pneumoniae and Haemophilus influenzae type b (Hib) are the most common causes of severe pneumonia in children in the developing world. In addition, a respiratory syncytial virus is the commonest viral cause of pneumonia that usually causes mild symptoms [2].

Despite overall hospital admissions for child pneumonia decreased by two folds since 2000, the disease continues to be the single largest infectious cause of mortality than any other infectious diseases such as diarrhea, malaria and AIDS killing one child every 39 s, 2,200 every day and 800,000 children under five every year [3, 4]. More than 900,000 children died from pneumonia, a preventable and treatable illness, in 2015 alone, accounting for 16% of under-five child mortality worldwide, with 3% of newborns. Africa and South-East Asia accounted for more than three-quarters of global pneumonia deaths in children younger than 5 years. Ethiopia along with India, Nigeria, Pakistan, and the Democratic Republic of the Congo contributed to the highest number of pneumonia deaths in children younger than 5 years accounting for 49% of global pneumonia deaths [3, 4].

Ethiopia has brought remarkable reduction in childhood morbidity and mortality through implementing various strategies. As part globally integrated management of childhood illnesses(IMCI) strategy, Ethiopia has been implementing integrated management of childhood illnesses (IMCI) at the health centre and hospital level, and integrated community case management at the community level [4]. In addition, the most current version of strategy focusing on the good health of the child has been started to be implemented to bring meaningful reduction of childhood diseases including pneumonia [5]. Despite such efforts, Ethiopia is the 5th leading country in pneumonia cases and deaths in countries with a high burden of the diseases in 2017 [6].

The child’s age, mother’s age, child’s sex, educational status of parents, family income, use of unclean biomass fuel, lack of opening windows in the kitchen or homes, overcrowding, parental asthma, household history of acute lower respiratory infections, malnutrition, lack of exclusively breastfeeding, and lack of zinc supplementation were all identified in previous studies as predictors of pneumoniae. Child caring practice of the families and comorbidity conditions such as diarrhoea, measles, acute upper respiratory infection (AURTI), and previous history of asthma were also some of the determinants of pneumonia [7–9].

Although studies are conducted in Ethiopia, the determinant factors for pneumonia differ in different societies and study populations [10, 11], s, due to variations in prevention practices of the community [10, 11], socio-economic and demographic inequalities [12],differences in uptake of pneumonia vaccination [13], poor infection prevention practices of healthcare professionals, lack of readiness of health posts, healthcare provider’s lack of awareness and knowledge about pneumonia [11] and so on. Taking the high burden of pneumonia and the variability of risk factors into account, this study aimed to identify the determinants of pneumonia among under-five children to inform healthcare providers, local healthcare administrators and programmers, stakeholders and policymakers for designing better prevention and control interventions for pneumonia. Furthermore, there is no previous study in the area that could show the determinants. Therefore, this study aimed to identify determinants of pneumonia among under-five children in South West Shewa Zone, Oromia Region, Ethiopia, 2021.

## Methods

### Study setting and design

We conducted a facility-based unmatched case control study from March 15 to April 30, 2021, in public hospitals found in South West Showa Zone of Oromia region. South West Showa Zone is located at 114 Km away from Addis Ababa (capital city of Ethiopia) and to the South West part of the country. The zone has a total population of 1,101,129, of which about 951,251 (86.3%) were rural residents. And children under the age of five accounted for 205,747 (18.6%) [14].We conducted the study in five public hospitals found in the zone.

### Source of cases and controls

All under-five children visited all hospitals’ pediatric outpatient departments, emergency outpatient departments, and maternity wards were the source population for cases and controls. Study populations were children who were 2 to 59 months of old, and who were attending the selected hospitals during the study period.

Cases were children who were in 2 to 59 months old and visiting hospitals’ pediatric, emergency, and maternity departments, and registered and diagnosed with pneumonia within the study period using the WHO’s Integrated Management of Newborn and Childhood Illness (IMNCI) guideline adopted by Federal Ministry of Health [15]. Controls were children in the same age range with cases who attended similar departments of each hospital, and registered and diagnosed as no pneumonia using the same IMNCI guideline used in identifying cases during the study period.

#### Operational definitions

##### Pneumoniae

is defined as all children who came with a compliant of cough, grunting or difficulty of breathing and age specific fast breathing or consolidation or infiltration that is found be pneumonia or severe pneumonia according to the clinical signs and symptoms among under five children according to classifications of the IMNCI guideline of the WHO [16].

##### Fast breathing

defined as 60 or more breaths per minute for children less than 2 months old, 50 breaths or more per minute for those 2–12 months old, and 40 breaths or more per minute for those 12–59 months old [16].

##### Family history of acute respiratory infection (ARI)

refers to a child in household with a history of pneumonia, bronchitis, ear infection, common cold, tonsillitis, or pharyngitis confirmed by a clinician during the 15 days prior to data collection.

##### History of low birth weight

for this study applies to if the child weighs < 2500 g at birth. [17]

##### Overcrowding

is defined by World Health Organization, as a situation in which more people are living within a single dwelling as compared to space so that movement is restricted, privacy is limited, hygiene is poor and disturbed sleep [18].

##### Full vaccination

includes all children who had obtained BCG (Bacillus Calmette Gue´rin vaccine) and OPV0 (oral polio vaccine at birth), pentavalent 1 (DPT-hepB-Hib [diphtheria, pertussis, tetanus, hepatitis B and Haemophiluss influenzae type b), OPV1, and PCV1 at 6 weeks; pentavalent 2, OPV2, PCV2 at 10 weeks; pentavalent 3, PCV3 at 14 weeks; and measles vaccine at 9 months.

### Inclusion and exclusion criteria

Children whose age ranged from 2 to 59 months were included in the study if children had lived in the Zone for at least six months to restrict the inclusion of children outside the study region to reduce bias in concluding the findings. Children with cardiac diseases, chronic respiratory diseases such as childhood asthma, cough lasting for more than 15 days, cough due to a recent history of aspiration of a liquid or foreign body, and caregivers who did not have any information about the child at the time of data collection were excluded from the study.

### Sample size determination

Sample size was determined using the two population proportion formula [19].

1 $$n = \left( {\frac{{r + 1}}{r}} \right)\frac{{\left( {\bar p} \right)\left( {1 - \bar p} \right){{\left( {{Z_\beta } + {Z_{\alpha /2}}} \right)}^2}}}{{{{\left( {{p_1} - {p_2}} \right)}^2}}}$$

The sample size were computed for all potentially significant predictors. Then, the factor, parental smoking gave the biggest size which was picked for our sample size calculation. The proportion of exposure in cases (p1) and controls ((p2)) had been determined for various predictors from the previous study [20]. Accordingly, the proportion of parental smoking in cases (p1) from the previous study was 25.9% and the proportion of parental smoking in controls (p2) was 14.3%. We used statistical normal distribution valueof Zα/2 at a 95% confidence interval (1.96); the desired power of the study (80%) (0.8) to calculate the minimum sample size. Based on the above assumptions, we calculated the sample size using EPI info version 7.2.2.6 software [21] and finally considered a 10% non-response rate. The ratio of cases to controls would be 1:1. Therefore, the minimum final sample size per group was 204 (204 cases and 204 controls).

### Sampling procedures

Five hospitals in the study area were included. In the five hospitals, physicians (GP or pediatrician) are responsible to manage the childhood illness. All are trained on IMNCI guideline. The sample size was allocated to each hospital proportionally to their average number of pneumonia cases attended per month based on the first six months of 2021 district health information system (DHIS2) report. Accordingly, the sample sizes for the hospitals of St. Lukas, Tulu B., Leman, Bantu, and Amaya were 144 (72 cases and 72 controls), 124 (62 cases and 62 controls), 52 (26 cases and 26 controls), 50 (25 cases and 25 controls), and 38 (19 cases and 19 controls), respectively. Until the required sample size was reached, children under the age of five who were diagnosed and reported with pneumonia/severe pneumonia were considered as the case in the study. One case for each control was selected from each similar unit/department consecutively. We randomly picked an odd number for cases and an even number for controls at the starting date of data collection as starting points in each facility. Then, we continued picking cases and controls consecutively until the allocated sample size was reached. Finally, interviewing of the mothers/care givers was done for both cases and controls at exit time in a separate room.

### Data collection tools and procedures

A study team prepared a structured questionnaire based on the study objectives by reviewing literatures relevant to the study [20, 22–28]. The questionnaire has four sections addressing potential determinants for pneumoniae in children under five years old. The sections include socio-demographic characteristics (such as age, sex, area of residence, occupation, income, educational status, and marital status), maternal-related factors (such as gravidity, childbirth space, smoking, knowledge on handling of domestic smoking, and antenatal care (ANC) utilization), child-related factors (such as birth weight, age, sex, immunization status, and breastfeeding practice, birth status of the fetus, co-morbidity, and supplementary feeding), and housing and environmental-related factors (family size, housing condition, cooking rooms, type of fuels used for cooking, source of light in the house, a house near to the street, household ventilating practices, hand washing practice, daycare centre/nursery school attendance practice, overcrowding and co-residence).

We prepared a questionnaire in English. Then, it was translated to “Afan Oromo” by a specialized and a fluent professional of the language, and then re-translated it to English to check for its consistency. We pre-tested the questionnaire in 5% of the sample before the study begins and revised it according to the feedback. We recruited and trained five data collectors who have bachelor’s degrees in nursing and two supervisors who have master’s degrees in public health. After we identified the study subjects as cases and controls by IMNCI trained physicians (GP or pediatrician), the data collector took them to a separate room for an interview. Finally, we made a record review to collect information like height, weight, and HIV status of the child. Completeness and consistency of data were checked by the principal investigator timely for feedback on each data collection day.

### Data processing and analysis

We entered using Epi- version 7.2.2.6 and checked its accuracy, consistency, outliers and missing values. We exported data to the SPSS version 23 statistical software package for analysis. We used descriptive statistics such as means, proportions, graphs and tables. To see the association between predictors and the outcome variable (pneumonia status of children aged 2 to 59 months), we calculated crude and adjusted odds ratios. Variables having a statistical association with the outcome variable at P-value ≤ 0.20 in the binary logistic regression were candidates for the multivariable logistic regression to control for confounders. We declared variables as potential independent predictors of pneumonia if they had a p-value of < 0.05 at 95% CI. The Hosmer and Lemeshow goodness-of-fit were used to test for model fitness before the actual interpretation of the findings.

## Results

### Socio-demographic characteristics of respondents

Out of 408 mothers/caregivers of the children, 398 (199 cases and 199 controls) were interviewed, yielding a response rate of 97.5%. The mean age of the respondent was 28.7 years with SD ± 6.9 years among cases and 29.2 years SD ± 6.3 among controls with a range of 17 to 48 years old. The mean age of children among the cases and controls was 15.3 and 19.7months with SD ± 14.4and 16.3 months, respectively. Out of the respondents, 58(29.1%) of mothers/caregivers among cases and 43 (21.6%) among controls were between 18 and 24 years of old. Among all under-five children included in the study, 60 (30.2%) of cases and 40 (20.1%) of controls were less than six months of age. About 60% of the cases and 47% of controls resided in the rural area. In cases and controls, the majority of the mothers interviewed were married. Table 1 shows the overall socio-demographic characteristics of respondents.

**Table 1:** Socio-demographic characteristics of respondents in South West Shewa Zone, Ethiopia, 2021

Variables	Category	Cases = N (%)	Control = N (%)
Age of mothers	< 18 years	12(6%)	5(2.5%)
	18–24 years	55 (27.6%)	55 (27.6%)
	25 years and above	132 (66.3%)	139(69.8%)
Residence	Urban	79 (39.7%)	106(53.3%)
	Rural	120 (60.3%)	93 (46.7)
Marital Status	Single	10 (5.0%)	6(3.0%)
	Married	169 (84.9%)	162(81.4%)
	Divorced and others	20(20.1%)	31(15.5%)
Mother’s/care giver’s education	No formal education	53(26.6%)	48(24.1%)
	Able to read and write	29 (14.6%)	35(17.6%)
	Primary (1–8)	50(25.1%)	59(29.6%)
	Secondary and above	67 (33.7%)	57(28.6%)
Mother’s /care giver’s occupation	House wife	101(50.8%)	95(47.7%)
	Merchant	32(16.1%)	37(18.6%)
	Student and Others	27(13.6%)	34(17.1%)
	Employee	39(19.6%)	33(16.6%)
Father’s education	No formal education	49(28.6%)	43(21.6%)
	Able to read and write	39(19.6%)	42(21.1%)
	Primary (1–8)	40(20.1%)	41 (20.6%)
	Secondary and above	71(35.7%)	73 (36.7%)
Father’s occupation	Farmer	105 (52.5%)	97 (48.7%)
	Merchant	31(15.6%)	25(912.6%)
	Others	37 (18.6)	44 (22.1)
	Employee	26 (13.1)	33 (16.6)
Family size	Less than five	92(46.2%)	101(50.8%)
	Five and above five	107(53.8%)	98 (49.2%)

### Child and maternal related characteristics

Of the total participants, 77 (38.7%) of cases and 41 (20.6%) of controls had breastfed for less than six months of life. However, 92 (46.2%%) of cases and 99 (49.7%) of controls started complementary feeding at six months of age. Regarding nutritional supplementation, in the majority of the cases, 148 (74.4%) and controls, 93(46.7%) had not taken Vitamin A in the six months before the data collection. Concerning vaccination status, 76 (38.2%) of cases and 114(57.3%) of controls were fully vaccinated while, 14(7%) and 6 (3%) of cases and controls have no history of vaccination, respectively. Regarding a child’s medical history, 98 (42.2%) cases and 21 (10.6%) controls had a history of a URTI in the 15 days before the data collection. Nine out of ten mothers in cases have a history of using ANC, compared to 14.6% of mothers in controls. Among the cases and controls, 139 (69.8%) of the children were raised by their mothers, while the remaining children were looked after by domestic help or family members. More than 72% and 60%% respondents among cases and controls have not trained/heard about handling of domestic smoking, respectively. Child and maternal- related characteristics are depicted in Table 2.

**Table 2:** Characteristics of respondents and their association with pneumonia among under-five children in South West Shewa Zone Hospitals, Ethiopia, 2021

Variables	Category	Cases; No(%)	Controls; No(%)	COR (95%CI)	AOR (95%CI)
**Socio-demographic**					
Age of mothers*	< 18 years	9(4.5)	3(1.5)	2.53 (0.87, 7.37)	
	18–24 years	58(29.1)	43 (21.6)	1.05 (0.62, 1.64)	
	25 years and above	132(66.3)	153(76.9)	1	
Residence*	Urban	79 (39.7)	106(53.3)	1	
	Rural	120 (60.3)	93 (46.7)	1.73 (1.16, 2.58)	
**Maternal and child**					
Child caregiver**	Mother	139(69.8)	165(82.9)	1	1
	House worker	60(30.2)	34(17.1)	2.10(1.30,3.38)	6.46(2.83,14.76)
Knowledge of domestic smoking*	Yes	30(15.1)	78(39.2)	1	
	No	169(84.9)	121(60.8)	2.27 (1.46–3.52)	
Mother’s use of ANC*	Yes	181(90)	178(84.9)	1.43(0.84, 2.42)	
	No	18(9)	21(10.6)	1	
Age of child*	< 6 moth	60(30.2)	40(20.1)	2.11(1.29, 3.45)	
	6,12 moth	58(29.1)	45(22.6)	1.81(1.12, 2.94)	
	> 12 moth	81(40.7)	114(57.3)	1	
Birth weight of child*	< 2.5 kg	17(8.5)	10(5.)	1.77 (0.79, 3.96)	
	≥ 2.5 kg	182(91.5)	189 (95	1	
Duration of breast feed**	< 6 month	77(38.7)	41(20.6)	3.40 (2.02, 5.71)	3.51(1.12,11.00)
	6–12 month	75(37.7)	73 (36.7)	1.86 (1.15, 3.01)	2.07(0.88,4.89)
	> 12 month	47(23.6)	85(42.7)	1	1
Immunization status of the child*	Fully vaccinated	76 (38.2)	114(57.3)	1	
	Vaccinated for age	62 (31.2)	47 (23.6)	1.98(1.23, 3.19)	
	Partially vaccinated	47(23.6)	32 (16.1)	2.20(1.29, 3.76)	
	Not vaccinated	14 (7.0)	6 (3.0)	3.50(1.29, 9.51)	
Vit A supplementation in the last 6 months**	Yes	51(25.6)	106 (53.3)	1	1
	No	148(74.4)	93 (46.7)	3.31(2.26,5.26)	3.56(1.58, 8.05)
Zink supplementation in the last 6 month*	Yes	19(9.5%)	60 (30.2)	4.09(2.33, 7.17)	
	No	180(60.5)	139 (69.8)	3.08 (1.94, 4.90)	
History of diarrhea in last 2 weeks*	Yes	79 (39.7)	35 (17.6)	1	
	No	120(60.3)	164 (57.7)	8.22(4.84,13.98)	
Child history of URTI in 2 weeks**	Yes	113(56.8)	21(10.6)	3.69(1.19,11.41)	9.66(4.69,19.87)
	No	68(43.2)	178(89.4)	1	1
**Housing and Environmental**					
Number of people sleeping in the same room**	One-two	22(11.1)	51(25.6)	1	1
	Three-five	151(75.9)	140(70.4)	2.50(1.44, 4.34)	2.90 (1.23, 6.84)
	> five	26(13.1)	8(4.0)	7.53(2.95,19.23)	3.88(1.02,14.77)
Availability of and or daily windows opening practice**	Yes	62(31.2)	118(49.3)	1	1
	No	137(68.8)	81(40.7)	3.22(2.13, 4.86)	2.56(1.21, 5.41)
Place of cooking*	In the living room	54(27.1)	26(13.1)	2.14(1.10, 4.13)	
	Kitchen attached to LR	110(55.3)	137(68.4)	0.83(0.49, 1.40)	
	Separate kitchen	35(17.6)	36(16.1)	1	
Place to keep child from cooking area*	On mother’s back	17(8.5)	8(4.)	3.52(1.46, 8.49)	
	Within 2 m	95(47.7)	47(23.6)	3.35(2.16, 5.19)	
	Kept > 2 m	87(43.6)	144(72.4)	1	
Source of fuels for cooking**	Wood/charcoal	149(74.4)	96(48.2)	3.90(2.47, 6.18)	3.02(1.41,6.46)
	Kerosene	13(6.5)	10(5)	3.27 (1.32, 8.10)	2.44(0.58,10.38)
	Electric	37(18.6)	93(46.7)	1	1
Source the of light in the house*	Wood	15(7.5)	5(2.5)	3.97(1.40,11.22)	
	Kerosene	63(31.7)	34(17.1)	2.45 (1.52, 3.96	
	Electric or solar	121(60.8)	160(80.4)	1	
Proper hand washing practice*	Yes	127(63.8)	142(71.9)	1	
	some times	67(33.7)	52(26.1)	1.45(0.94, 2.24)	
	No	5(2.55)	4(2.0)	1.41(0.37, 5.39)	
Presence of latrine *	Improved	20(10.1)	34(17.1)	1	
	Not improved	64(32.2)	68(34.2)	1.60(0.84, 3.06)	
	Traditional	93(46.7)	88(44.2)	1.80 (0.96, 3.36)	
	No latrine	22(11.1)	9(4.5)	4.16(1.60,10.77)	

Note: ** Shows independent variables significantly associated with dependent variables in a multivariable logistic regression analysis at P = < 0.05.

Note: * shows factors associated with presence of pneumonia at p, values ≤ 0.2.

### Housing and environmental related characteristics

Concerning housing conditions, 62(31.2%) of the cases and 118 (49.3%) of the controls reported that they had opened windows on a daily bases. Additionally, 54 (27.1%) of respondents among cases and 26 (13.1%) controls were cooking foods in the living room. Similarly, 95 (47.7%) and 47 (23.6%) of the child were kept at a 2-meter distance from the cooking area during cooking foods among cases and controls, respectively. Moreover, nearly three in four cases and half of the controls had used wood and/or charcoal as a source of fuel for cooking. Only 20(10%) of the cases and 34(17%) of the controls had improved latrine facilities and 22(11%) cases and 9(4.5%) of controls had no latrine facilities. Table 2 shows the housing and environmental characteristics of the participants.

### Factors associated with presence of pneumonia in under five children

As shown in Table 2, socio-demographic characteristics, child, parental, housing and environmental related factors were tested for their association with status of pneumonia among under-five children in a binary logistic regression analysis. Those eligible variables for multivariable logistic regression were tested for association with the outcome variable. Accordingly, seven independent predictors of pneumonia in children under the age of five were identified as a result of multivariable logistic regression: duration of breastfeeding, vitamin A supplementation, history of URTI, number of people sleeping in the same room, respondents’ propensity to open windows, fuel source for cooking, and family child care practice.

Compared to children who had been breastfed for 6 months or more, infants who had been breastfed for less than 6 months had a 3.5 times higher risk of developing pneumonia [AOR:3.51, 95%CI:(1.12,11.00)]. When compared to children who had no history of URTI in the previous two weeks, those with a history of URTI were 9.6 times more likely to develop pneumonia[AOR:9.66, 95%CI:(4.70,19.87)]. Children who were usually cared for by house workers or relatives had 6.4 times higher odds of contracting pneumonia than those who were mostly cared for by their moms or parents [AOR:6.46, 95%CI:(2.83, 14.76)]. Children from homes where wood/charcoal was utilized as a source of cooking fuel had a nearly 2 fold greater risk of contracting pneumonia [AOR: 3.02, 95%CI :( 1.41, 6.46)]. Children who had not taken vitamin A supplements in the previous six months were more than 3.5 times more likely to acquire pneumonia when compared to their counterparts [AOR:3.56, 95%CI:(1.58,8.05)]. When compared to their counterparts, children from households with no windows and/or no daily opening practice had 2.5 times the risk of having pneumonia [AOR:2.56 95%CI: (1.21,5.41)]. When compared to those sleeping with fewer than two people per room, children who slept in a room with 3 to 5 people had a 2.9 times higher risk of contracting pneumonia[AOR: 2.90, 95%CI:(1.23, 6.84)]. Furthermore, those who slept in a room with > 5 people had a 3.9 times higher risk of contracting pneumonia [AOR: 3.90, 95%CI :( 1.82, 14.77)].

## Discussion

Our study highlighted that non-exclusive breastfeeding, lack of vitamin A supplement, child’s history of upper respiratory infections, parental care other than the mothers, room overcrowding, not opening of windows while cooking (poor ventilation practice) and using a less clean source of fuel for cooking (wood and or charcoal) were statistically significant predictors of pneumonia in children under the age of five.

In our finding, non-exclusive breastfeeding (children who had not been breastfed for six or more months for our study) practice increased the risk of developing pneumonia in children under the age of five when compared to those who practice exclusive breastfeeding with their children. Our finding is consistent with the studies from Ethiopia [20, 29], other African countries and other low-income countries; India, Indonesia and Brazil [30–32]. However, our finding is not in line with the studies in Ethiopia [7–9, 33], and East African countries [34]. As studies witnessed breast milk is the best source of nearly complete nutrients and boosts immunity (maternal IgM and IgG and lymphocytes) [35] that help resist infection including pneumonia in the child. We do not believe that the consistency or inconsistency of our findings with others is not primarily due to differences in the above inherent advantages of exclusive breastfeeding but contextual breastfeeding practices across communities. Breastfeeding practice highly varies with complex factors like hygiene and sanitation practices of the community (awareness and practice on how to feed a child hygienically and poor sanitation warehouses (availability, accessibility and safety of water, access and affordability of detergents, household and environmental contaminants of food and water and so on). In addition, maternal factors of lactation, maternal and or parental lack of awareness and skill of appropriate breastfeeding, and lack of support from community healthcare workers like health extension workers in the context of Ethiopia. Hence, our finding implies that the problem of inappropriate breastfeeding practices for the prevention of a child from infectious diseases including childhood pneumonia still needs strengthening the current community-based strategies (ICCM &IMNCI) and integration of the current community-based nutrition (CBN) into those programs. Our findings show that the study region is far from targets of exclusive breastfeeding which is among important lists of prevention strategies in WHO’s Global Action Plan for Pneumonia to reducing mortality and morbidity of children due to infectious diseases including pneumonia [36]. Furthermore, the study implies lags behind the WHO feeding recommendations in the LMICS context [37]. Moreover, further studies need to be conducted to search for contextual child-friendly exclusive breastfeeding interventions for both rural communities (typical nature of our study population) and urban communities.

Our study also revealed that children who had previously experienced acute upper respiratory tract infection had a higher risk of acquiring pneumonia than those who had not. This finding is consistent with studies reported from southern Ethiopia [7, 8], Northern Ethiopia [8, 9, 33] and other East African countries at large [34].Upper respiratory tract infections are highly communicable through air-born, direct, and indirect contacts and lead to invasion of the lung by the specific pathogens that trigger an immune response and cause inflammation [2]. In addition, acute respiratory infections including URTIs alter the structure and function of the lower respiratory tract that may cause lower respiratory tract infections including pneumonia either by creasing invasion of the lower respiratory tract (LRT) with other pathogens to cause secondary infections or by the progressive invasion of LRT with the same pathogens causing the ARTIs [38]. Another possible explanation could be due to the fact that the key interventions to reduce and or prevent URTIs include immunization against specific pathogens, early diagnosis and treatment of URTIs, improvement in nutrition like vitamin A supply and hygiene and environmental interventions are not still being implemented at the required level in the study area. Our finding may also imply that lack of leadership support and the need for local contextual interventions for proper integration of acute respiratory infection preventions into the current ICCM or IMNCI and other child survival strategies due to other competing needs like coronavirus disease − 19. Especially, the problem would be very serious in the case of a rural community, which is the typical characteristic of our study population. Such challenges are also recently documented in a global review [39].

Regarding micronutrient supplementation, our study indicated that children who had not received vitamin A in the six months prior to data collection had a higher risk of developing pneumonia than children who had received vitamin A. Our study is in line with studies done in other parts of Ethiopia [8, 40] Rwanda [41] and Java [42]. The role of vitamin A is still an essential micro-nutrient intervention in the growth and development of respiratory epithelial cells and lung tissue and improving a good immunity against acute respiratory infections including pneumonia [41]. On top of this, supplementation of vitamin A to a child would have an irreplaceable advantage. Because studies indicate that children in low- and middle-income countries are at increased risk of vitamin A deficiency due to various reasons like low liver stores at birth, decreased vitamin A in breast milk due to maternal malnutrition, lack of adequate breastfeeding, decreased absorption and increased losses due to recurrent gastrointestinal infections [41]. Our study implies equitable supplementation of vitamin A to children would have averted a significant proportion of children from being infected with pneumonia. Therefore, the integration of micro-nutrient supplementation including vitamin A into the current child survival strategies and intervention in the study areas as well as in the country should be re-evaluated and strengthened to reduce the risk of acute respiratory infections including pneumonia.

According to our findings, children mostly looked after by house workers or relatives were at higher odds of developing pneumonia as compared to children who were carried by their parents. This finding agrees with a study done in the Amhara region, Ethiopia and Northwest Ethiopia [23, 24] and Eastern Africa [34]. This might be due to the fact that house workers or relatives may not be responsible for child care as their parents such as keeping child’s self-hygiene, feeding the child on time and other child care activities. Hence, the study implies that parents who gave childcare responsibility to their housemaids and or relatives need to make a close follow-up in all matters related to respiratory infections including pneumonia. Furthermore, contextual studies and interventions regarding parental care of a child should be in place.

Our study also identified environmental predictors of pneumonia in children under the age of five: source of fuel, overcrowding and availability and or opening of the window.

The current study also revealed that children from parents using wood/charcoal/animal dung as a source of fuel for cooking were more likely to develop pneumonia when compared with children from parents who use electricity as a source of fuel for cooking. The finding was supported by findings from the studies in Southern [7] and North Western Ethiopia [33, 43] and Eastern Africa [34], Northern Ethiopia [9], and other low and middle-income countries like India and Indonesia [30, 44]. This is due to using unclean as a source of fuel like wood and or charcoal results in a release of very fine particulate matter and or major air pollutants like carbon monoxide which may get into the lung and aggravate the inflammation that had already been initiated by pathogens causing pneumonia [45]. Furthermore, it impairs the function of pulmonary alveolar macrophages and epithelial cells, which will increase the likelihood of pulmonary infections including pneumonia [45, 46]. Hence, our study suggests the need to promote and design clean fuel and how to adapt the already available unclean source of fuel through local engineering methods like proper chimney system for the kitchen and adequate ventilation by like opening the window. The finding still calls for additional local interventions through collaborating the health extension with agricultural extension programs in the study area to alleviate the problem.

Our findings show that a larger number of children who share the same room were more likely affected by pneumonia. The finding agrees with a similar study conducted in other parts of Ethiopia [28, 47] and Eastern Africa [34], Northern Ethiopia [9] and Brazil [48]. Despite the difficulties of avoiding overcrowding in low-income countries like Ethiopia where there is higher average family size, ventilation practices and other household sanitation conditions have to be well promoted. Finally, our study identified that absence of windows and or practice of not opening windows on daily bases was significantly associated with the occurrence of pneumonia. Our finding is in agreement with studies from Southern Ethiopia [49], Oromia region [50] and outside Ethiopia [51] all show poor home ventilation increases the probability of pneumonia infection among under-five children. Studies documented that improved household air quality through ventilating a house or room continuously with fresh air can reduce pneumonia cases [52]. However, our findings suggest the need to further promote the health benefits of household ventilation practice and research local interventions to improve the ventilation system to avert air-born acute respiratory infections thereby reducing pneumonia in children under the age of five.

There was no significant difference between cases and controls with regard to age the of child, child birth weight, child immunization status, lack of Zink supplementation, child history of diarrhoea, maternal ANC utilization status, mothers’ knowledge of domestic smoking, source of light in house, hand washing practice, and latrine unitization despite eligible for the multivariable logistic regression model. However, variables like child malnutrition status [7, 8, 33], immunization status [9, 34] and history of diarrhoea [9] were statistically significant predictors of pneumonia under the age of five. This could be either contextual differences between the study areas and study design or the factors are equally distributed among cases and controls that may not clearly show that they are not predictors and need additional studies for further aversion of the determinants of pneumonia among children under five.

## Limitations of the study

Diagnosis of pneumonia was based on clinical WHO IMNCI classification guidelines, which may introduce misclassification bias. The recall bias could also be another limitation that may affect some associations between the determinants and the outcome of the study.

## Conclusion

The study identified lack of exclusive breastfeeding for six months, child history of URTI, lack of vitamin A supplementation, caregiver of a child, use of wood and or charcoal as a source of fuel, sleeping with three or more persons in a room and absence of window and/or no daily opening practice as predictors of pneumonia among under-five children.

Our findings may imply the need to avert persistent determinants through implementing lists of prevention strategies in WHO’s Global Action Plan for Pneumonia to reducing mortality and morbidity of children due to infectious diseases including pneumonia in the healthcare system of the study area in particular, the region in general. Hence, we suggest the need to re-strengthen the already available interventions (i.e. iCCM, IMNCI and CBN) and check whether these programs are being implemented as planned by focusing on identified determinants by the study. In addition, exclusive breastfeeding, sustainable supplementation of vitamin A, safe parental care related to respiratory infections, adaptation and design of clean sources of fuel for cooking, ventilation practices and adequate ventilation, and avoiding overcrowding should be promoted to support and prove in interventions by integrating into the healthcare system.

Moreover, studies need to be conducted to search for contextual child-friendly exclusive breastfeeding interventions, contextual challenges of integration of acute respiratory infection preventions into the current ICCM, IMNCI and other child survival strategies and new interventions to avert persistent determinants of pneumonia.

## Data Availability

All the data required are available in the manuscript. But, if the datasets generated and/or analyzed in the current study are required, reasonable requests to access these datasets would be directed to the correspondent author as the dataset contains key identifiers such as medical record numbers of the children.

## Abbreviations

ALRTI: Acute Lower Respiratory Tract Infection
ARI: Acute Respiratory Tract Infection
IMNCI: Integrated Management of newborn and Childhood Illness
DHIS: District health information system
CBN: Community based nutrition
ICCM: Integrated community case management
HIV: Humman immune deficiency virus
AOR: Adjusted odds ratio
SPHMMC: Saint Paul’s Hospital Millennium Medical College
UNICEF: United Nations Children’s Fund
URTI: Upper Respiratory Tract Infection
WHO: World Health Organization
